# Thyroid hormone deficiency worsens outcomes in vaccinia virus infection

**DOI:** 10.1128/jvi.01294-25

**Published:** 2025-11-20

**Authors:** Laura Notario, Erika Guerrero-Espinosa, Manuel Nistal, Pilar Lauzurica, Ana Aranda, Susana Alemany

**Affiliations:** 1 Centro Nacional de Microbiología, Instituto de Salud Carlos III 38176, Majadahonda, Madrid, Spain; 2 Departament de Metabolismo y Enfermedades Inmunes. Instituto de Investigaciones Biomédicas “Sols-Morreale”, Consejo Superior de Investigaciones Científicas-Universidad Autónoma de Madrid 16722, Madrid, Spain; 3 Departamento de Anatomía, Histología y Neurociencia, Facultad de Medicina, Universidad Autonoma de Madrid 70695 , Madrid, Spain; Dartmouth College Geisel School of Medicine, Hanover, New Hampshire, USA

**Keywords:** immune response, vaccinia virus, thyroid hormones, alveolar macrophages, Sirtuin 1

## Abstract

Intranasal inhalation of the vaccinia virus in mice leads to acute lung infection followed by peripheral organ damage. Our findings demonstrate that hypothyroidism significantly exacerbates disease severity. Hypothyroid mice exhibit higher disease scores, elevated lung viral loads, and more extensive tissue damage in both the lungs and peripheral organs. Notably, only hypothyroid mice reach the experimental endpoint, underscoring their heightened vulnerability. Hypothyroid mice display a defective splenic immune response, with no amplification of T and B lymphocytes, but the increased susceptibility to vaccinia virus infection also persists in hypothyroid lymphocyte-deficient Rag2^-/-^ mice. Prior to infection, hypothyroid mice show a reduced pool of lung alveolar macrophages, and lung viral loads are significantly elevated in these animals as early as 1 day post-infection, suggesting that an impaired innate immune response is involved in their increased susceptibility to vaccinia virus. Indeed, transfer of primary alveolar macrophages into the alveolar macrophage-deficient lungs of hypothyroid mice significantly alleviates disease symptoms. In euthyroid mice, circulating thyroid hormone levels decrease during infection, a well-documented response in sepsis and critical illness, known as non-thyroidal illness syndrome. Further highlighting the link between thyroid hormones and immune defense, we show that SRT1720, a Sirtuin 1 activator, reduces thyroid hormone levels and also worsens vaccinia infection when administered to euthyroid mice. In summary, our study reveals that hypothyroidism aggravates vaccinia virus infection. While the role of thyroid hormone decline in various diseases remains a topic of debate, our results suggest that thyroid hormones play a protective role in viral pulmonary infections.

**IMPORTANCE:**

Vaccinia virus serves both as a recombinant vaccine platform and as a model for studying human smallpox and monkeypox infections, which are associated with high mortality rates. Here, we show that hypothyroidism in mice aggravates the severity of vaccinia virus infection due to a deficient splenic immune response and to a marked reduction in lung alveolar macrophages, a key cell population in defense against respiratory pathogens. Intratracheal administration of primary alveolar macrophages improves disease symptoms during the early phase of infection in hypothyroid animals. Hypothyroidism also impairs the amplification of splenic lymphocytes, which play a key role in defense against viral infection. Furthermore, vaccinia virus infection reduces thyroid hormone levels in euthyroid mice, a phenomenon named “non-thyroidal illness syndrome” that often occurs in septic patients, suggesting that, in the context of viral pulmonary infections, thyroid hormone replacement might be a useful therapeutic option.

## INTRODUCTION

Vaccinia virus (VACV), cowpox virus, and variola virus, which causes smallpox, are members of the Poxviridae family, a double-stranded DNA virus family that can cause disease in humans (1). VACV was used to eradicate smallpox, is currently used as a vector for immunization against other pathogens, and is extensively employed as a model for human smallpox and monkeypox, which have a high death rate (2). In mice, inhalation of VACV infects the lungs, where it replicates exponentially, resulting in lung inflammation and damage. Subsequently, the infection also affects other organs (3). VACV infection clearance is first controlled by the innate immune response, with alveolar macrophages (AMs) representing the first barrier against lung viral infection (4, 5). However, if VACV infection is not resolved, a powerful adaptive immune response by lymphocytes is required for recovery from respiratory VACV infection (3, 4). In the orchestrated response to viral infection, the production of pro-inflammatory cytokines and chemokines also plays an important role (5).

Thyroid disorders profoundly impact patients’ lives on a chronic basis and pose a substantial global public health burden. These conditions, encompassing both hypothyroidism and hyperthyroidism, span a spectrum from subclinical to severe and can manifest with a wide range of symptoms, affecting nearly every bodily system. Studies indicate a hypothyroidism prevalence of approximately 1.3% in the general population, which increases to 2%–15% in pregnant women and 7%–20% in individuals over 65 years of age, with higher incidence observed in specific iodine-deficient areas (https://www.who.int/data/nutrition/nlis/info/iodine-deficiency [6, 7]). The thyroid hormones, triiodothyronine (T3), the active hormone, and thyroxine (T4), its precursor, are key regulators of growth, metabolism, and energy homeostasis (8). The link between the immune and endocrine systems is increasingly well established, with evidence of a functional crosstalk between the thyroid hormones and the immune system (reviewed in references 9, 10), which may contribute to pathophysiological conditions, including sepsis, inflammation, autoimmune diseases, and viral infections (11). Abnormally low plasma concentrations of thyroid hormones often occur in septic patients in the absence of thyroidal disease. This phenomenon, known as the “euthyroid sick syndrome” or “non-thyroidal illness syndrome” (NTIS) (12, 13), is correlated with illness severity and outcome. During sepsis, the thyroid axis is affected, with reduced pituitary release of the thyroid-stimulating hormone and peripheral inhibition of the conversion of T4 to T3, the active hormone (12). NTIS has been regarded as an adaptive metabolic response in an attempt to ameliorate metabolic stress by lowering metabolic activity (12, 14). However, whether the decrease in circulating thyroid hormones is protective or detrimental for an effective immune response is currently under debate and may depend on pathogen type, severity, and affected organ (12). Thus, we and others have shown that COVID-19 patients with low free T3 levels showed a worse prognosis and higher levels of the COVID-19 severity markers, together with a metabolomic cluster indicative of a high ketogenic profile (15, 16), while hypothyroidism in mice confers tolerance to cerebral malaria, caused by the *Plasmodium berghei* parasite. This is mimicked by activation of the NAD^+^-dependent deacetylase Sirtuin 1 (SIRT1) (17). SIRT1 is a crucial regulator of metabolic processes in response to changes in nutrient availability, thereby controlling energy homeostasis and the metabolic state (18, 19), and orchestrating immune and inflammatory responses during infection (20).

Given the prevalence of thyroid disorders in specific populations, we aimed to elucidate the role of thyroid hormones within the context of viral infection. We have analyzed the effect of hypothyroidism and hyperthyroidism on the response to intranasal VACV infection. Our results show that hypothyroid mice are more susceptible to the infection, presenting higher disease scores, elevated lung viral titers, and enhanced lung and peripheral organ damage. Treatment of euthyroid mice with a SIRT1 activator lowers thyroid hormone levels and mimics the effect of hypothyroidism, suggesting the involvement of this metabolic enzyme in the control of viral load. Circulating thyroid hormone levels regulate the expansion of splenic immune cells during VACV infection. Rag2^-/-^ hypothyroid mice, with a disrupted adaptive immune system, also exhibit increased susceptibility to VACV, suggesting that a deficient innate immune response is also involved in this increase. Before infection, hypothyroid mice show a markedly reduced AM population, along with higher viral load and more disease symptoms as early as 1 day post-infection (p.i.). Intratracheal transplant of primary AMs alleviates disease symptoms after infection, especially during the first days. In conclusion, hypothyroidism causes weakened splenic and innate immune responses to VACV, increasing disease severity.

## RESULTS

### Circulating thyroid hormones after VACV infection

**Fig 1:**
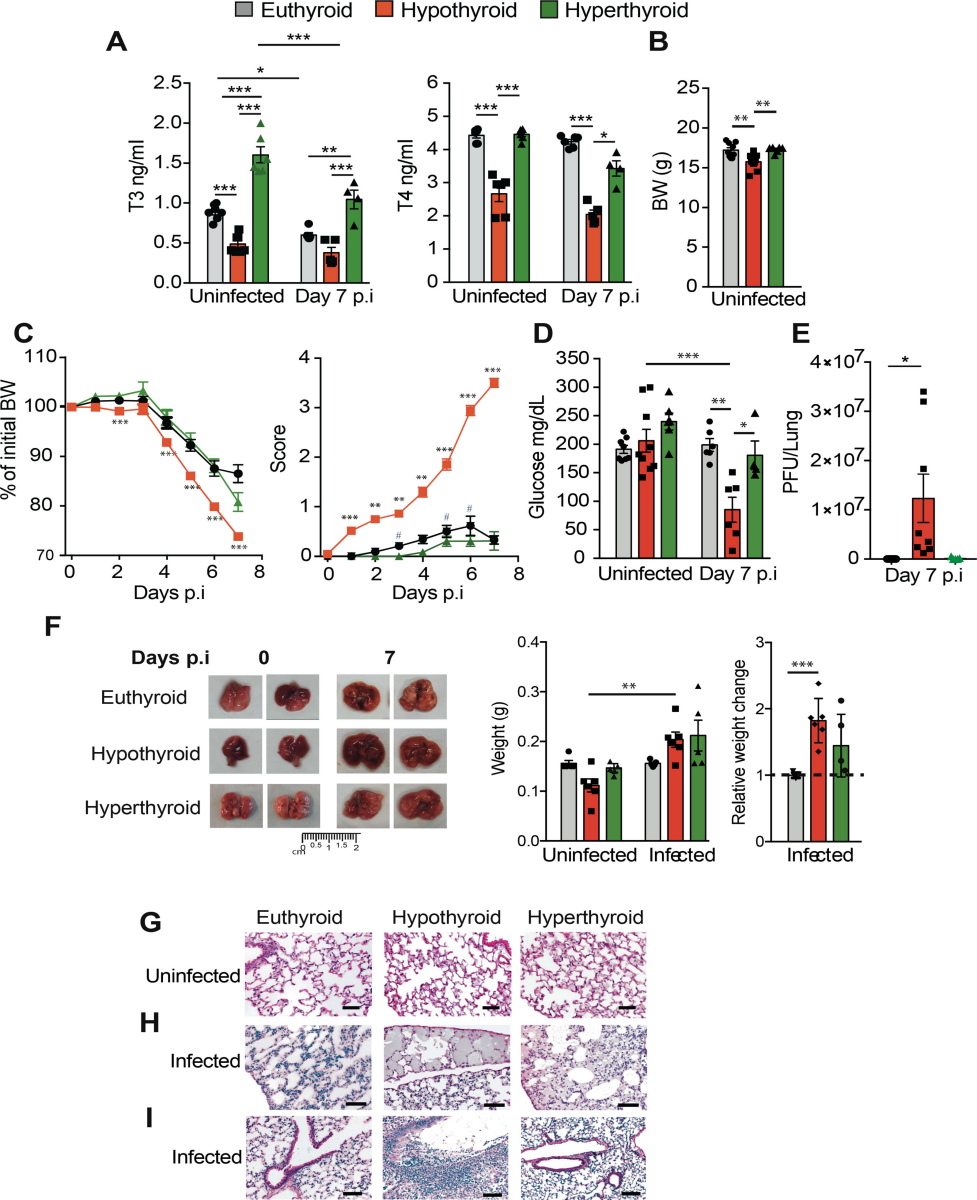
Thyroid hormones regulate sensitivity to VACV infection. (**A**) T3 and T4 levels measured at days 0 and 7 after intranasal infection with 5,700 PFU of VACV per gram of body weight in euthyroid, hypothyroid, and hyperthyroid mice. (**B**) Body weight at time 0. (**C**) Left panel: body weight evaluated daily after infection. Data are expressed relative to the initial body weight shown in panel B. Right panel: disease score during infection. (**D**) Circulating glucose levels at days 0 and 7 after infection. (**E**) Viral titers in lungs collected at day 7 p.i. (**F**) Lungs were excised at days 0 and 7 p.i. The left panel shows representative images of the lungs. The weight of the lungs is shown in the middle panel. The increase of lung weight at day 7 versus day 0 of infection is illustrated in the right panel. (**G**) Representative H&E staining of the lungs of non-infected euthyroid, hypothyroid, and hyperthyroid mice. (**H**) H&E staining of the lungs at day 7 p.i., showing exacerbated edema in the hypothyroid mice. (**I**) Bronchopneumonia and alveolar necrosis are present in the infected hypothyroid mice but not in euthyroid or hyperthyroid mice. Scale bars 50 µm. *P < 0.05, **P < 0.01, and ***P < 0.001.

In humans, both hyperthyroidism and hypothyroidism exist on a spectrum from subclinical to severe (9, 10). Prior to infection, hypothyroid mice were generated by feeding them an iodine-deficient diet containing an anti-thyroidal drug for 4 weeks. These mice exhibited a 50% reduction in circulating T3 levels and also a 25% decrease in T4 compared to euthyroid mice on the day of the infection (Fig. 1A). In contrast, oral thyroid hormone treatment increased circulating T3 levels, resulting in hyperthyroid mice that showed 1.8-fold higher levels. Because T3 treatment depletes pituitary thyrotropin and blocks endogenous thyroid secretion, mice were also given T4 to maintain normal circulating levels of this hormone. These observed variations in circulating T3 in these mouse models represent moderate hypothyroidism and hyperthyroidism, respectively, with a much higher incidence than severe thyroid disorders, particularly in the Western world. These three types of mice were intranasally infected with VACV. Upon inhalation in mice, VACV targets the lungs, where it undergoes an exponential replication phase for approximately the first 3 days. This is followed by a plateau with a high load that typically lasts for approximately another 3 days, with values dependent on the MOI. Subsequently, the infection’s resolution or progression is contingent upon the prevailing experimental conditions (3, 4, 21). At day 7 p.i., hypothyroid mice did not show a further significant decrease of both T3 and T4, but euthyroid mice showed a 32% reduction in T3, while T4 levels were not reduced (Fig. 1A). After infection, T3 levels were reduced in hyperthyroid mice. The decrease in T3 levels observed at 7 day p.i. with constant iodine and T3 plus T4 hormone supply in euthyroid and hyperthyroid mice, respectively, indicates that VACV infection alone is sufficient to induce NTIS in these mice.

### Increased sensitivity of hypothyroid mice to VACV infection

Uninfected hypothyroid mice display reduced body weight, as expected (17), while hyperthyroid mice have normal body weight (Fig. 1B). From day 4 after intranasal VACV infection, all animals started to lose weight, but the relative weight loss in the hypothyroid mice was significantly more marked than in euthyroid or hyperthyroid animals (Fig. 1C), suggesting an increased susceptibility to VACV infection in hypothyroidism. By day 7 p.i., euthyroid mice had lost 7% of their initial body weight, hypothyroid mice 25%, and hyperthyroid mice 13%. Due to this significant weight loss in hypothyroid mice, they were considered to have reached their experimental endpoint, were humanely euthanized, and the experiment was terminated. At the time of termination, these hypothyroid mice showed a high disease score of 3.5 ± 0.10, out of a possible maximum score of 4, whereas euthyroid and hyperthyroid animals showed much lower scores of 0.3 ± 0.09 and 0.3 ± 0.11, respectively. This increased score in hypothyroid mice was also consistently present throughout the infection. Symptoms were detectable as early as day 1 (Fig. 1C), when no symptoms were observed in control animals. Conversely, hyperthyroid mice displayed a small reduction in disease score compared to euthyroid mice, which was statistically significant at some time points. Low circulating levels of glucose are a severity marker of sepsis (22). At day 7 p.i., significant hypoglycemia was observed only in hypothyroid mice, which displayed a 60% decrease in their baseline glucose values. In contrast, euthyroid mice showed no decrease, while hyperthyroid mice exhibited a 25% decrease (Fig. 1D). Most importantly, at this time, hypothyroid mice had significantly higher lung VACV titers than euthyroid mice, with 12.3 ± 4.3 million more PFU per lung. Hyperthyroid mice, in comparison, did not present statistically significant differences in viral titers compared to the euthyroid group (Fig. 1E).

Hemogram analysis showed that red blood cell counts were not significantly affected at day 7 of VACV infection, and that platelet numbers were similarly increased across all groups of infected mice. In contrast, the number of circulating leukocytes was reduced upon VACV infection and was significantly lower in the hypothyroid mice both prior to infection and p.i. Hypothyroid mice showed a 35% reduction in the number of leukocytes prior to infection compared to euthyroid mice. This value further decreased by 42% at the endpoint. This reduction largely reflects the lower number of lymphocytes, the more abundant circulating white blood cell population. Neutrophil counts were induced to similar values in all groups, although only in euthyroid mice was the increase statistically significant, and no significant differences in monocyte numbers were observed (Fig. S1A). As the expression and secretion of cytokines and chemokines regulate inflammation and initiate antiviral responses, we also determined the effect of hypothyroidism and hyperthyroidism on circulating cytokine responses to VACV. In serum collected prior to infection, no appreciable differences were observed among euthyroid, hypothyroid, and hyperthyroid mice. However, by day 7 p.i., several cytokines, including IFN-γ, IL-6, CCL2, and CXCL10, showed a stronger inflammatory response in hypothyroid mice compared to euthyroid controls, exhibiting approximately 1.5- to 2.5-fold higher values in the hypothyroid group, whereas CCL5 levels were higher in the hyperthyroid group, indicating that hyperthyroid mice mounted a potent antiviral or T cell-mediated response compared to the hypothyroid and euthyroid animals (Fig. S1B).

### Increased lung pathology after VACV infection in hypothyroid mice

As the lung is the primary organ affected after VACV inhalation, we analyzed changes in lung weight and morphology at day 7 p.i. Lungs are smaller (Fig. 1F) and hypoplastic (Fig. 1G) in uninfected hypothyroid mice. However, after infection, a strong increase in lung weight was observed in these animals, while changes were weaker or not significant in euthyroid and hyperthyroid mice. This suggests an exaggerated inflammatory reaction in hypothyroid mice. Infected hypothyroid mice displayed strong lung edema, which was only focal or absent in the other groups, and abundant inflammatory lymphocyte and polymorphonuclear cell infiltration, very dense in the hypothyroid mice, was observed in the interstitial space of all infected animals (Fig. 1H). Furthermore, clear signs of necrotizing bronchopneumonia, bronchial exudate, and bronchiolar necrosis were only detected in infected hypothyroid mice at day 7 p.i. (Fig. 1I).

We also investigated the circulating levels of organ damage markers. No changes in creatine phosphokinase, urea, or creatinine were detected after infection, although basal creatinine levels were lower in the uninfected hyperthyroid mice. However, and according to the higher viral titers and pathology in hypothyroid mice, a clear increase of the liver transaminases alanine aminotransferase, aspartate aminotransferase, and of the general tissue damage marker lactate dehydrogenase levels was observed in the infected hypothyroid mice, while these parameters were little affected in the euthyroid and hyperthyroid groups (Fig. S2A). These results suggest that liver function could be altered in infected hypothyroid mice. Indeed, only in these mice did VACV infection cause a significant loss of liver weight (Fig. S2B), suggesting again that hypothyroid mice are more susceptible to VACV infection and that peripheral organ pathology might be detected in these animals. Liver histology (Fig. S2C) showed that VACV infection caused portal and parenchymal immune cell infiltration and Kupffer cell hyperplasia in all groups, but only hypothyroid mice displayed cell death as shown by the presence of eosinophilic Councilman bodies, which represent apoptotic or necrotic hepatocyte cell fragments. Moreover, the stronger periodic acid-Schiff (PAS) staining in non-infected hypothyroid liver, indicative of a higher glycogen content (23), was lost at day 7 p.i. (Fig. S2D). This is compatible with the observed hepatic weight reduction and the hypoglycemia, indicating an increased energy input from glucose to fight infection in hypothyroid animals. Hyperthyroid livers showed the expected reduction of glycogen before infection (24, 25), but no further decrease was found at day 7 p.i.

### Splenic immune host response to VACV infection in hypothyroidism and hyperthyroidism

**Fig 2:**
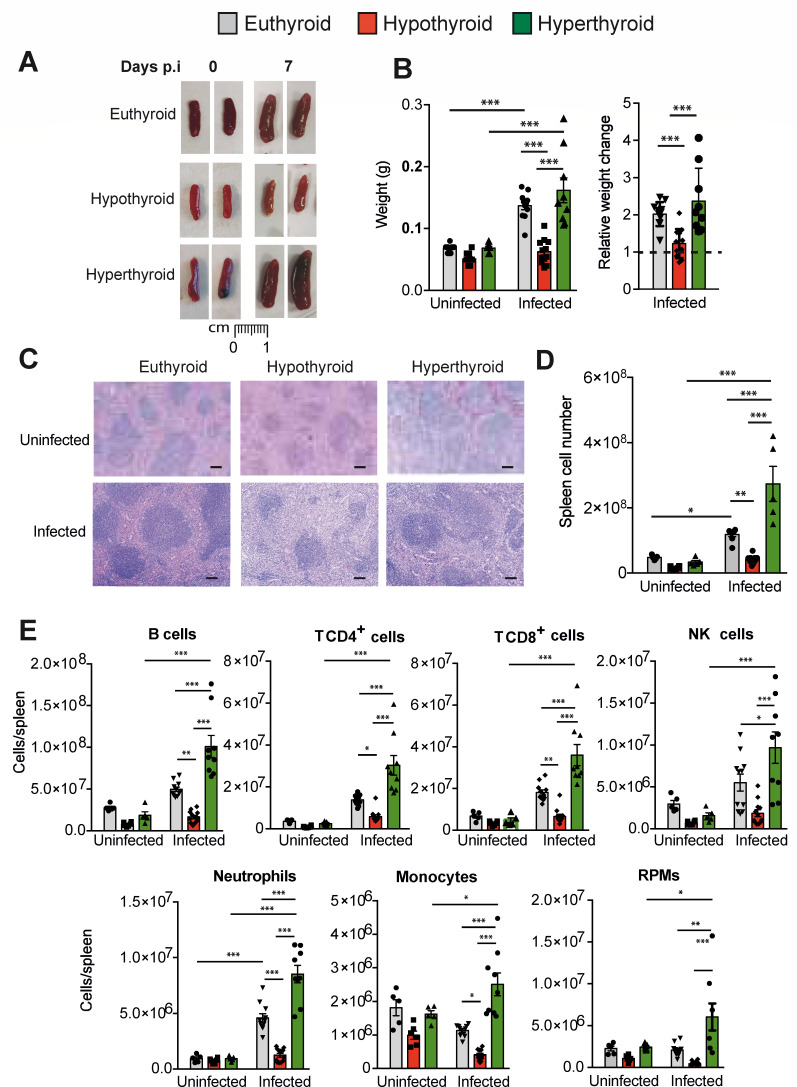
Hypothyroid mice display a reduced splenic response after VACV infection. (**A**) Representative images of the spleens of euthyroid, hypothyroid, and hyperthyroid mice at days 0 and 7 p.i. with 5,700 PFU of VACV per gram of body weight. (**B**) Spleen weight at day 0 (uninfected) and at day 7 p.i. (infected) of mice indicated in panel **A**. The left panel represents the absolute spleen weight, and the right panel shows the splenic weight in the infected mice with respect to the corresponding uninfected splenic weights. (**C**) Representative H&E images of the spleens at days 0 and 7 p.i. Scale bars: 100 µm. (**D**) Spleen cellularity was analyzed at 0 and 7 days p.i. in euthyroid, hypothyroid, and hyperthyroid mice. (**E**) Number of splenic B cells (B220^+^CD3^-^), T CD4^+^ cells (CD3^+^CD4^+^B220^-^), T CD8^+^ cells (CD3^+^CD8^+^B220^-^), natural killer (NK) cells (NKp46^+^B220^-^CD3^-^), neutrophils (CD11b^+^Ly6G^+^), RPMs (CD11b^+/low^Ly6G^-^F4/80^high^), and inflammatory monocytes (Ly6C^+^CD11b^+^Ly6G^-^) was determined by flow cytometry at days 0 and 7 after infection. Gating strategy is shown in Fig. S8. *P < 0.05, **P < 0.01, and ***P < 0.001.

The spleen, as a secondary lymphoid organ, plays a role against infection. Different types of splenic leukocytes are amplified to respond effectively to infection, which results in splenomegaly (3, 4). This was indeed observed in euthyroid and hyperthyroid mice, where spleen weight was significantly higher at day 7 p.i. than prior to infection. However, hypothyroid mice showed a reduced splenic response with only a minor increase of spleen weight upon infection (Fig. 2A and B). Of note, uninfected hypothyroid mice also displayed spleen hypotrophy in homeostasis when compared with euthyroid and hyperthyroid animals (Fig. 2A and B), as previously described (17, 26). Histological examination indicated an important increase in the splenic lymphoid nodules, often presenting germinal centers in euthyroid mice, even more marked in hyperthyroid mice, while hypothyroid spleens were atretic, showing smaller and fewer white pulp nodules (Fig. 2C). Correspondingly, at day 7 p.i. of VACV, euthyroid mice, and particularly hyperthyroid mice, showed a marked increase in the number of total splenic immune cells (Fig. 2D), including lymphocytes (Fig. 2E), with an essential role in the defense against VACV infection (3, 4). In contrast, hypothyroid mice did not exhibit this expansion of immune cells and, prior to infection, already showed a marked reduction in the number of total splenocytes, as well as in the different immune cells tested, including B and T lymphocytes, red pulp macrophages (RPMs), neutrophils, and monocytes, with respect to uninfected euthyroid and hyperthyroid spleens (Fig. 2E). All these results indicate that hyperthyroid mice are able to build up a very strong splenic immune response to VACV, whereas splenic hypothyroid cells do not expand in response to the viral infection. These data agree with Varedi et al. (27), showing that isolated splenic lymphocytes from hyperthyroid mice display a greater proliferative capacity than their counterparts from euthyroid mice after *in vitro* exposure to antigens from HSV-1, a virus that belongs to the Herpesviridae family.

### SIRT1 activation reduces thyroid hormone levels and increases susceptibility to VACV infection

**Fig 3:**
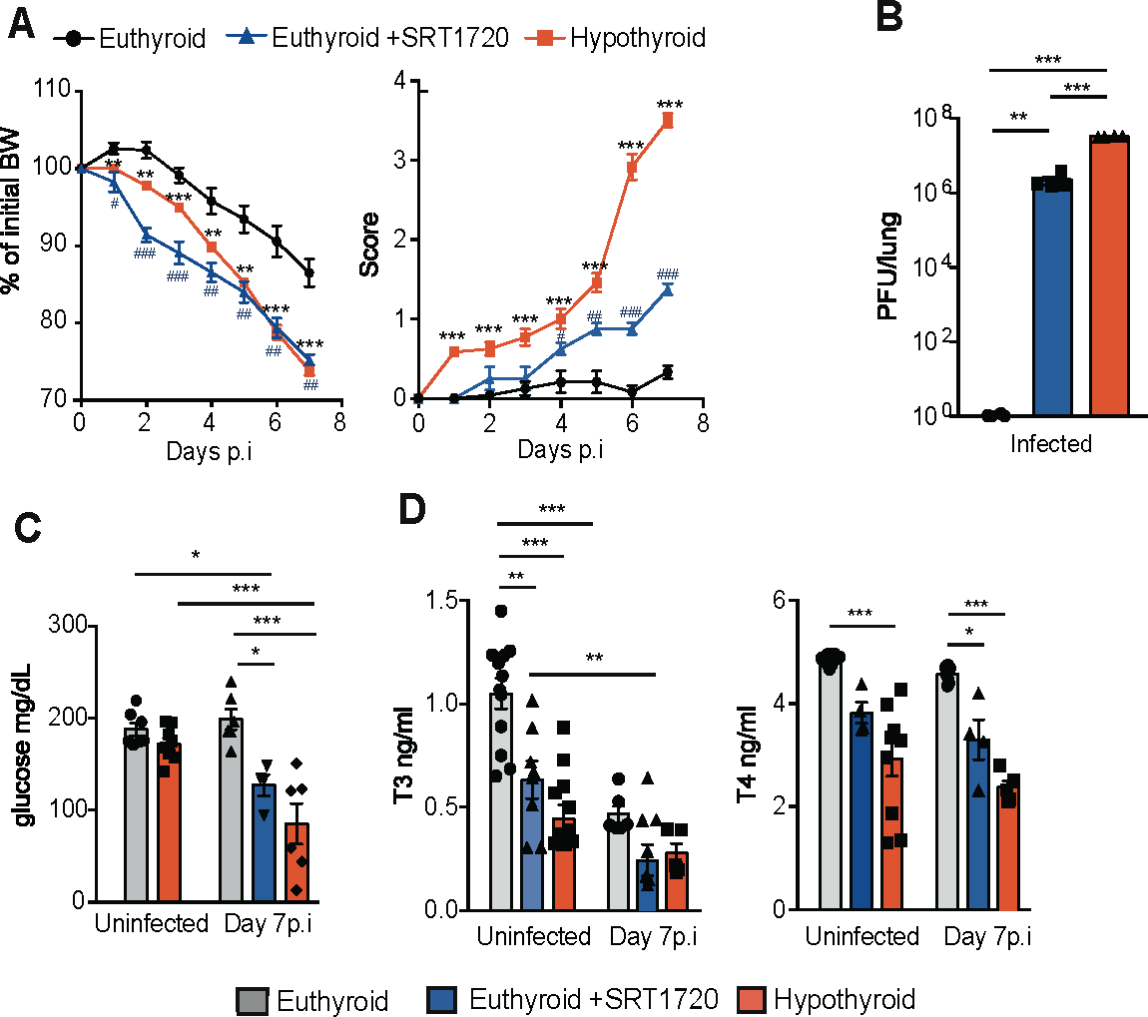
SIRT1 activation reduces circulating thyroid hormone levels and increases susceptibility to VACV infection. (**A**) Euthyroid mice were treated daily with vehicle or SRT1720 from the day of infection with 5,700 PFU of VACV per gram of body weight. A group of hypothyroid mice was inoculated in parallel with the virus. Body weight loss (left panel) and disease score (right panel) were recorded from day 0 to day 7 p.i. (**B**) Lungs of infected mice were excised at day 7 for determination of viral titers. (**C**) Circulating glucose levels at days 0 (uninfected) and 7 p.i. (**D**) Circulating T3 and T4 levels measured at days 0 and 7 after infection. *P < 0.05, **P < 0.01, and ***P < 0.001.

SIRT1 has an important regulatory function in host defenses following infection (20), and mimics the effects of hypothyroidism on cerebral malaria (17). We then examined the effect of SRT1720, a SIRT1 activator, on the response to VACV in euthyroid mice. Daily treatment with SRT1720 from the day of infection accelerated body weight loss and resulted in a significant increase in disease score, although not as marked as that induced by hypothyroidism (Fig. 3A). The number of circulating leukocytes, lymphocytes, and neutrophils was also lower in infected euthyroid mice treated with the SIRT1 activator, again mimicking the effect of hypothyroidism (Fig. S3A). Euthyroid mice treated with SRT1720 during the course of the disease also displayed a reduced splenic weight after infection, although much less marked than that found in hypothyroid mice (Fig. S3B), and statistically significant differences in counts of splenic populations were only observed for RPMs, which were reduced, and monocytes, which were increased by the treatment (Fig. S3C). In contrast to the results in hypothyroid mice, the SIRT1 activator was not able to alter liver weight (Fig. S3B). Concomitantly, the peripheral organ damage markers alanine aminotransferase, aspartate aminotransferase, and lactate dehydrogenase were elevated in infected hypothyroid mice, but this increase was not observed in infected euthyroid mice treated with SRT1720. No significant differences in creatine phosphokinase, urea, and creatinine were observed between the different groups (Fig. S3D).

Importantly, SIRT1 activation also elicited a significant increase of lung viral titers, which were in between the values found in the hypothyroid and euthyroid mice (Fig. 3B), and the same occurred with glucose levels, closer to hypothyroid mice than to euthyroid mice (Fig. 3C). The similar effects of hypothyroidism and SRT1720 on VACV infection prompted us to examine whether the SIRT1 activator alters thyroid hormone levels. A single daily administration of SRT1720 for 7 days significantly reduced T3 levels in euthyroid mice. However, this reduction was not as pronounced as the T3 deficiency observed in hypothyroid mice, which had been treated with a hypothyroid diet for 4 weeks. Moreover, VACV infection caused a further decrease of T3 levels in SIRT1-treated mice, which were no longer different from those found in hypothyroid animals. Circulating T4 levels were also between the values obtained in euthyroid and hypothyroid mice both before and after VACV infection (Fig. 3D). These results show that SIRT1 activation causes hypothyroidism, aggravating VACV infection, and corroborate that the virus causes NTIS, affecting primarily T3 levels.

### Hypothyroidism increases susceptibility to VACV infection in lymphocyte-deficient mice

**Fig 4:**
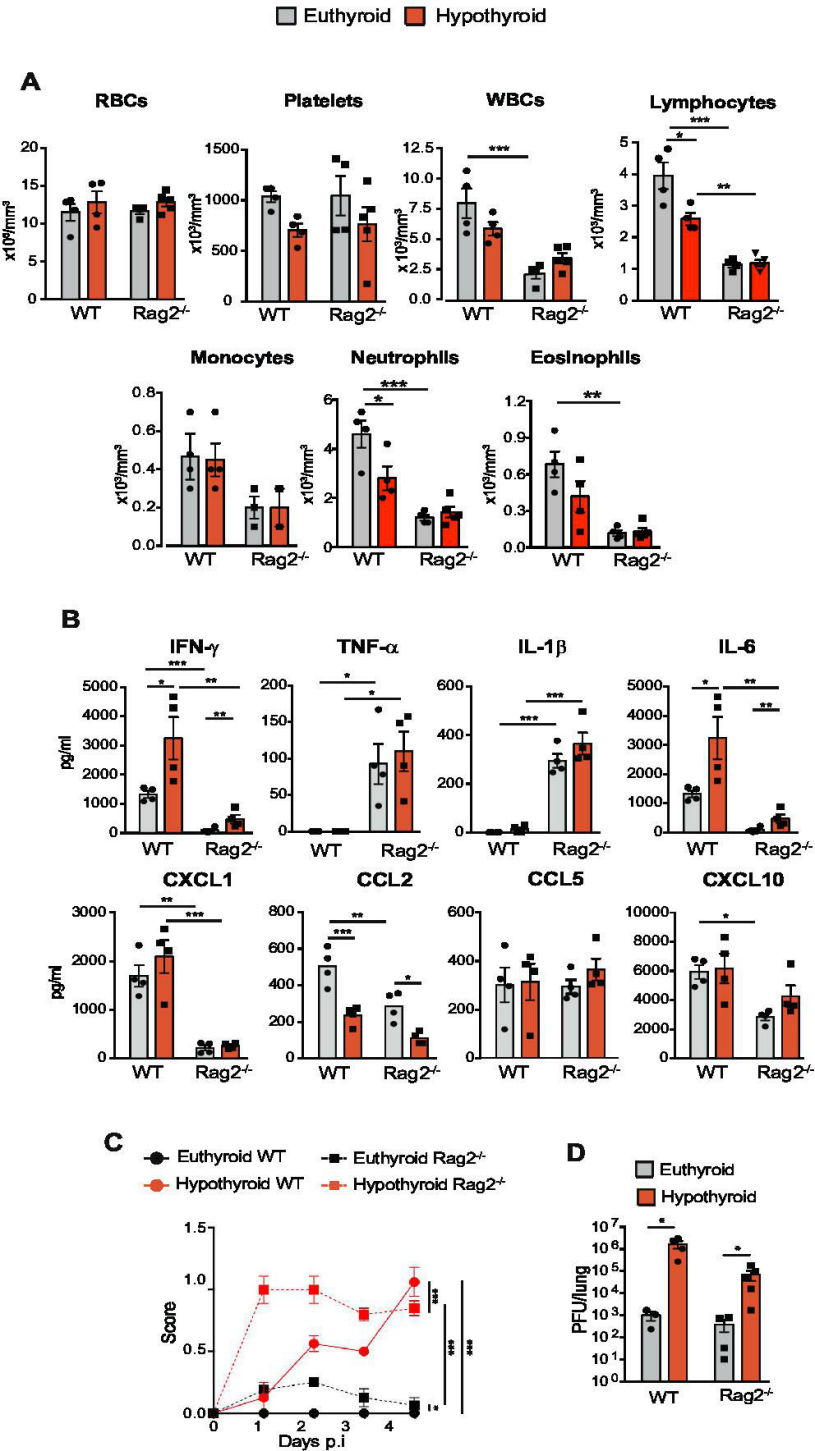
Hypothyroidism increases susceptibility to VACV infection in Rag2^-/-^ mice. (**A**) Euthyroid and hypothyroid wild-type (WT) BALB/c and Rag2^-/-^ BALB/c mice were intranasally infected with 5,700 PFU of VACV per gram of body weight, and a hemogram was performed at day 4 p.i. RBC, red blood cells; WBC, white blood cells. (**B**). Circulating cytokines in the same groups of animals. (**C**) Disease score was determined during the 4 days of infection (*n* = 8). (**D**) Lung viral titers at day 4 p.i. in euthyroid and hypothyroid WT and Rag2^-/-^ mice. *P < 0.05, **P < 0.01, and ***P < 0.001.

Considering the impaired amplification of hypothyroid splenic immune cells in response to VACV infection, we next analyzed this infection in euthyroid and hypothyroid Rag2⁻^/^⁻ mice along with euthyroid and hypothyroid wild-type (WT) mice. Rag2⁻^/^⁻ mice lack T and B cells, which constitute the adaptive immune system (28). As expected, these mice showed, 4 days after infection, a reduction in the number of different types of circulating white blood cells with respect to WT mice, while red blood cell and platelet counts were similar, with no significant differences between euthyroid and hypothyroid mice (Fig. 4A). Similarly, spleen cellularity, including mainly T and B lymphocytes, was very markedly reduced in Rag2^-/-^ mice with respect to WT mice (Fig. S4). In contrast, euthyroid Rag2^-/-^ mice, but not hypothyroid Rag2^-/-^ mice, presented elevated NK cells and monocytes after infection. RPMs showed a higher increase in Rag2^-/-^ mice independently of the thyroidal status and a similar number of neutrophils with respect to the WT animals. These differences in the number of hematopoietic cells support the different patterns of circulating cytokine expression after infection, with Rag2^-/-^ mice showing reduced levels of IFN-γ, IL-6, CCL2, CXCL1, and CXCL10, but an important increase of TNF-α and IL-1β levels. Hypothyroidism increased IFN-γ and IL-6 levels in both WT and Rag2^-/-^ mice, indicating a higher inflammatory state, while simultaneously reducing CXCL10 (Fig. 4B). Importantly, euthyroid Rag2^-/-^ mice showed accelerated clinical manifestations and a higher disease score than their euthyroid WT counterparts; and hypothyroidism further exacerbated disease symptoms (Fig. 4C). Hypothyroid mice also exhibited higher virus titers in their lungs (Fig. 4D). These results suggest that hypothyroidism confers a poor response to VACV infection, regardless of its impact on the adaptive immune system.

### Deficient innate immune response to VACV infection in hypothyroid mice

**Fig 5:**
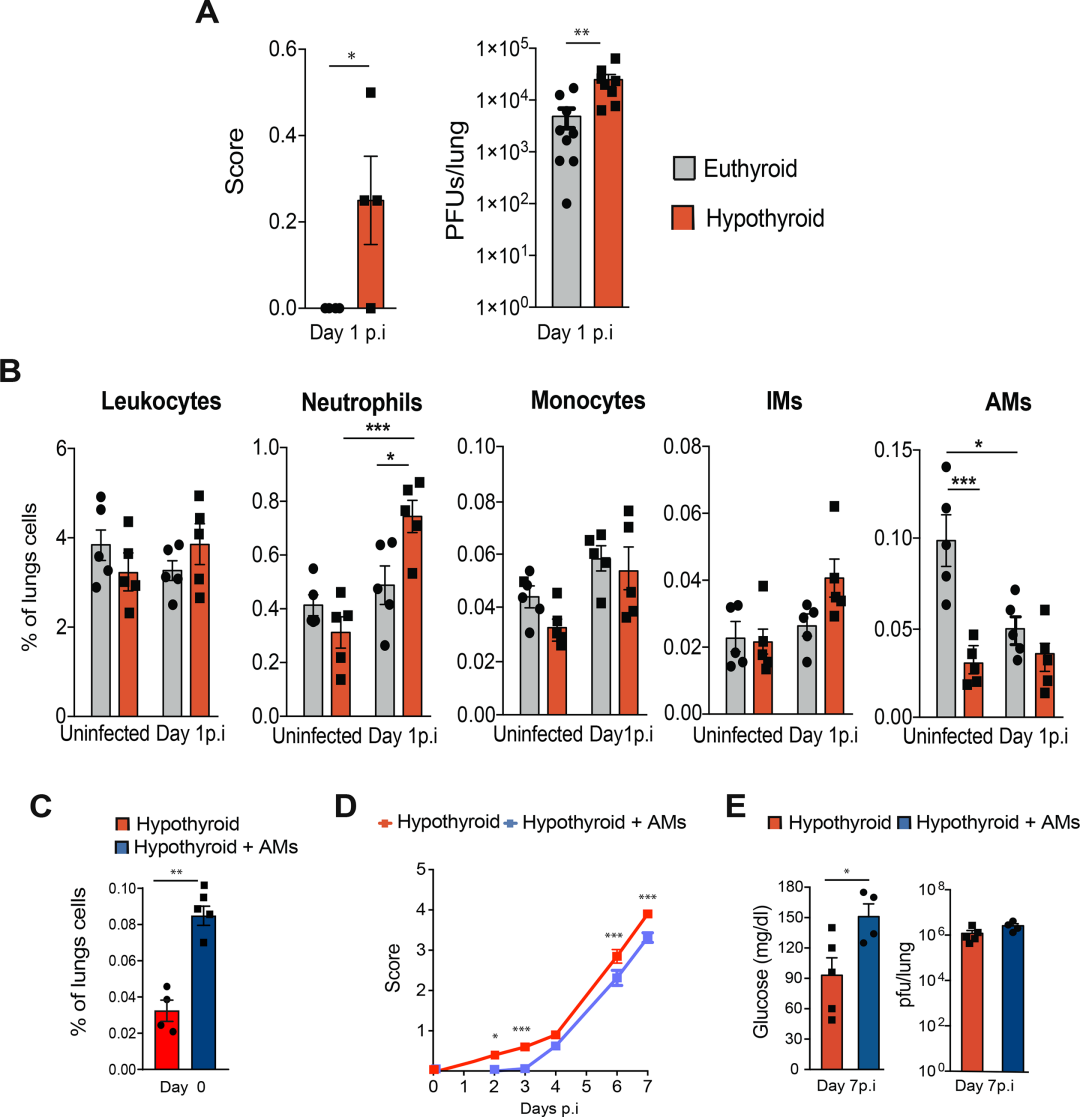
The innate immune response is involved in the increased susceptibility of hypothyroid mice to VACV infection. (**A**) Euthyroid and hypothyroid mice were intranasally infected with 5,700 PFU of VACV per gram of body weight and disease score (left panel) and lung virus titers (right panel) determined 1 day p.i. (**B**) Percentage of lung CD45^+^ cells, neutrophils (CD45^+^Ly6G^+^), monocytes (CD45^+^Ly6G^-^CD11c^low/+^CD11b^+^MHCII^-^CD64^low/-^), interstitial macrophages (IMs, CD45^+^Ly6G^-^CD11c^low^CD11b^+^MHCII^-^CD64^+^SYGLEC^-^), and AMs (CD45^+^Ly6G^-^CD11c^+^CD11b^+^MHCII^-^CD64^+^SYGLEC^+^), was assessed by flow cytometry in both groups of mice at days 0 (uninfected) and 1 p.i. The gating strategy is shown in Fig. S9. (**C**) Percentage of AMs in hypothyroid mice 1 day after the transfer of either 20 µL of PBS or 0.5 million normal primary AMs in PBS on the day of the infection. (**D**) Disease score in mice described in panel **C** following intranasal infection with 5,700 PFU of VACV per gram of body weight was followed for 7 days p.i. (*n* = 5,4). (**E**) Circulating glucose levels and lung viral titers determined at day 7 p.i. in mice described in panel **D**. *P < 0.05, **P < 0.01, and ***P < 0.001.

We then analyzed lung viral titers in euthyroid and hypothyroid mice 1 day after intranasal VACV infection, before activation of the adaptive immune response. Hypothyroid mice, but not euthyroid mice, showed early symptoms of infection, and significantly increased viral titers were observed in their lungs. These findings suggest that a deficient innate immune system may exacerbate disease severity in hypothyroid mice following VACV infection (Fig. 5A). One day after infection, the hemogram is still normal (Fig. S5A), but circulating TNF-α, IL-1β, and CCL2 were markedly lower in infected hypothyroid mice with respect to euthyroid mice, while CXCL10 levels were higher (Fig. S5B). Flow cytometry analysis revealed no difference in the percentage of hematopoietic cells in the lungs of euthyroid and hypothyroid mice prior to infection and at day 1 p.i. Among myeloid cells, the percentage of neutrophils was similar before infection and at day 1 p.i.; however, hypothyroid mice exhibited more than a twofold increase in their percentage, a change not observed in euthyroid mice (Fig. 5B), indicating an exacerbated damage (29). The percentage of monocytes and interstitial macrophages was comparable between the euthyroid and hypothyroid mice, but AMs, critical as the first barrier against viral pulmonary infection and with a key role in initiating the local antiviral innate immune response (29, 30), showed a marked decrease in hypothyroid lungs prior to infection (Fig. 5B). AMs undergo cell death during exposure to pathogens (31). Indeed, at 1 day p.i., euthyroid mice showed a strong reduction in the number of AMs, almost equaling the numbers of hypothyroid AMs, which were similar before and after infection.

In a proteome array with 99 cytokines, chemokines, and other ligands, lungs from hypothyroid mice showed, before infection, changes in the expression of 19 proteins, 14 downregulated and 5 upregulated, with respect to the euthyroid lungs. GM-CSF, with a key role in AM maintenance (32–34), was barely detected in both euthyroid and hypothyroid lungs (Fig. S6A), and further analysis of this cytokine by flow cytometry indicated that hypothyroidism does not affect GM-CSF expression (Fig. S6B), suggesting that other factor(s), besides GM-CSF, control(s) the number of AMs.

Interestingly, splenic RPMs, which like AMs also develop early in mice (35), are also diminished in uninfected hypothyroid mice (Fig. 3), but bone marrow macrophages, which are derived from hematopoietic stem cells and maintained by different mechanisms (36), showed similar numbers in femurs of hypothyroid and euthyroid mice (Fig. S7). Before infection, bone marrow B cells were reduced, and T cells were increased in hypothyroid mice, while the number of neutrophils and NK cells was similar in hypothyroid and euthyroid mice (Fig. S7).

Transferring AMs is a novel therapeutic strategy under preclinical investigation for various lung diseases, including infections (37–39). To determine whether increasing the number of AMs in hypothyroid mice improved their capacity to resolve intranasal VACV infection and to discern the relevance of AM deficiency and lymphocyte responsiveness in the inability to resolve VACV infection in these mice, we intratracheally transferred 0.5 million normal primary AMs or PBS into uninfected hypothyroid mice, as previously described (38, 39). This transfer increased the percentage of AMs in their lungs by approximately 2.5-fold 1 day post-transfer, which was also the day of infection (Fig. 5C) and almost reached the percentage observed in euthyroid mice prior to infection (Fig. 5B). Notably, after infection, symptoms were reduced in hypothyroid animals that had been transferred AMs, with no detectable disease symptoms when innate immune cells dominated the immune response (Fig. 5D). By day 7 p.i., the disease score was still lower in hypothyroid mice transferred with AMs, and the circulating glucose levels were higher (Fig. 5E), indicating a better metabolic state (22). However, both types of mice showed similar PFU titers in lungs at this time point (Fig. 5E). These findings indicate that transferring AMs into the lungs of AM-deficient hypothyroid mice prior to infection, while improving some parameters during the course of the disease, is not sufficient to rescue the euthyroid phenotype, likely because it does not fully restore normal immune function.

## DISCUSSION

The Poxviridae family includes several viruses of medical and veterinary importance, and VACV can be used as a live recombinant vaccine against many different diseases (1). Therefore, it is necessary to define the determinants of the host response to these viruses in the respiratory tract, the main route of infection. We show here that thyroid hormones play a very important role in the overall severity of the disease. Hypothyroid mice exhibit more marked weight loss and stronger disease symptoms than euthyroid or hyperthyroid mice after intranasal instillation of VACV. Significantly, hypothyroid mice display substantially higher viral titers in the lungs and increased pulmonary pathology. According to these data, it has been shown that following a 3 day intraperitoneal infection with HSV-1, spleens from hyperthyroid mice have a lower titer than spleens of euthyroid mice, while the highest titers are found in the spleens of hypothyroid mice (40). After intranasal VACV infection, if the acute lung infection is not resolved, the infection’s course can lead to peripheral organ injury. In hypothyroid mice, we observed hepatic damage and strong hypoglycemia, which were not present in euthyroid or hyperthyroid mice. The hypoglycemia in hypothyroid mice correlates with the increased depletion of glycogen in the liver, indicating high metabolic stress (22).

SIRT1 activation has been proposed as a mechanism for the treatment of several diseases (41). However, activation of SIRT1 during VACV infection increases disease severity and lung viral load. These effects may be related to a reduction in circulating thyroid hormone levels. The degree of hypothyroidism caused by treatment with the SIRT1 activator, SRT1720, is less intense than that observed in our hypothyroid animals, which were fed a low-iodine diet containing an anti-thyroidal drug for 1 month prior to infection, but still sufficient to increase susceptibility to VACV infection.

Interestingly, deiodinase 3 knockout mice, which show central hypothyroidism with low circulating levels of T4 and T3 (42), display significantly higher bacterial load in the lung than wild-type mice after infection with *Streptococcus pneumoniae*, corroborating that low thyroid hormone levels are also associated with increased bacterial load in the respiratory tract (43). Hypothyroid mice also have a more severe acute inflammatory lung damage than euthyroid mice in a model of ventilator-induced pulmonary injury (VIPI). Moreover, administration of T3 to deiodinase 2 knockout mice, which also exhibit reduced T3 and T4 levels, reduces damage, dampening the inflammatory response to VIPI (44). Clinically, hypothyroidism is prevalent in patients with idiopathic pulmonary fibrosis and has been associated with unfavorable prognosis (45), and it has been proposed that thyroid hormones may represent a potential therapy for the disease (46). Also, we and others have observed that COVID-19 patients with low thyroid hormone levels have a poorer prognosis than patients with higher hormone levels (15, 16). Taken together, these results indicate a positive action of thyroid hormones in the control and outcome of various respiratory diseases.

In addition to VACV infection, other experimental models associated with lung injury display NTIS (43, 44). Whether the reduction in circulating thyroid hormone levels associated with NTIS is beneficial or detrimental to the host immune response is still under debate (12). Our results suggest that in the context of viral pulmonary infection, thyroid hormones may exert protective properties, leading to an improved immune response. Thus, further studies are needed to determine whether thyroid hormone supplementation, either alone or as adjunctive therapy, might be favorable for the outcome of respiratory infections. However, hypothyroidism is beneficial in cerebral malaria (17), reinforcing the idea that NTIS may be advantageous or detrimental for a proper immune response, depending on the disease type, pathogen, and affected organ (12).

Hypothyroidism displays deficiencies in the innate as well as in the adaptive immune response to VACV infection. Hypothyroid mice show no adaptive immune response of splenic lymphocytes on day 7 post-infection. On the other hand, at day 1, when only the innate immune system is sensing the infection, hypothyroid mice show a higher degree of infection, and according to these data, RAG2^-/-^ mice lacking adaptive immune cells are also more sensitive to VACV infection. AMs are the predominant resident phagocytic cells in the lungs that respond to inhaled pathogens in the respiratory tract, initiate the local innate immune response (29, 30), and maintain the functional integrity of lung epithelium. In this context, depletion of AMs contributes to an accumulation of myeloid cells, mainly neutrophils, in the lungs after VACV infection, which likely contributes to more severe disease in response to VACV infection (29). We observed a striking decrease in the number of AMs in uninfected hypothyroid lungs. Concurrently, both WT and RAG2^-/-^ hypothyroid mice developed higher disease scores from the infection’s onset, during which the innate immune system governs the immune response. After VACV infection, different leukocyte populations are amplified in the spleen to effectively resolve the disease (3, 4). Euthyroid and hyperthyroid mice show clear signs of splenic stimulation following VACV infection, whereas hypothyroid mice show a significantly reduced splenic response. However, this is not a rigid rule, as enhanced amplification of splenocytes has been observed in hypothyroid mice compared to euthyroid mice in experimental cerebral malaria caused by *Plasmodium berghei* (17). This suggests that circulating thyroid hormones control the splenic immune response in a pathogen-dependent manner.

In hypothyroid mice, the transfer of AMs significantly improved the disease score, an effect that was most pronounced during the first days of the infection. Hypothyroid mice also displayed higher glucose, an indicator of less metabolic stress, but were not able to resolve VACV infection as euthyroid mice did. The transfer of AMs to hypothyroid mice allows AMs to reach similar levels to those observed in euthyroid mice. This improves the first days of infection, when innate immune cells, including AMs, are primarily responsible for the response. However, this transfer does not overcome the deficient immune response observed when cells of the adaptive immune system take over the response. The intrinsic thyroid state of lymphocytes modulates their proliferative capacity, and thyroid hormones do not require the generation of other signals to mediate this effect. Indeed, isolated lymphocytes with different thyroid states show different proliferation rates in response to different signals (9, 27).

In conclusion, in moderately hypothyroid mice, the defective immune response during VACV respiratory infection leads to increased lung damage, elevated circulating markers of peripheral tissue injury, low glucose levels, and the development of a non-resolving disease state. Therefore, thyroid hormone substitutive therapy should be considered for specific populations, such as pregnant women and individuals over 65 years, during respiratory viral infections.

## MATERIALS AND METHODS

### Mice and treatments

Female C57BL/6, BALB/c, and Rag2^-/-^ BALB/c mice were bred and housed under pathogen-free conditions at the animal facility of the Instituto de Salud Carlos III, Madrid, Spain. Unless otherwise indicated, all experiments were performed in C57BL/6 mice. To induce hypothyroidism, 4- to 5-week-old female mice were fed an iodine-deficient diet containing 0.15% of the anti-thyroidal drug propylthiouracil (E15551-04, Sniff) for 4 weeks before VACV infection. Euthyroid animals were fed with the same diet without propylthiouracil and supplemented with potassium iodide to contain 1.15 mg/kg (E15552-24, Sniff). Both diets were maintained until the end of the experiments. Hyperthyroid mice were fed the control diet and were made hyperthyroid by adding T4 (25 ng/g of mice; IRMM468, Sigma-Aldrich) and T3 (95 ng/g of mice; T-2877, Sigma-Aldrich) in the drinking water from 14 days before infection until the end of the experiment. Drinking water was changed every other day. Euthyroid mice were injected i.p. daily starting on the day of infection with the SIRT1 activator, SRT1720 (HY-15145, Med Chem Express) at a dose of 20 mg/kg in 100 µL as previously described (17). SRT1720 was dissolved in 10% DMSO (D2438, Sigma-Aldrich) and 20% 2-hydroxypropyl-β-cyclodextrin (H5784, Merck) in PBS. Considering that hypothyroid mice have a lower weight, mice were inoculated intranasally with an MOI of 5,700 PFU per gram of body weight of the Western Reserve strain of VACV. The virus was grown in CV1 cells cultured in DMEM supplemented with 10% fetal calf serum, 2 mM L-glutamine, 100 U/mL penicillin, 100 µg/mL streptomycin, and 5 µM β-mercaptoethanol, and virus titer was determined by plaque assay, as previously described (47, 48).

### Lung VACV titer assay

The viral load in the lungs was measured by plaque-forming assay. Mice were sacrificed at the times indicated in the experiments, and lungs were harvested and stored at −80°C in 0.5 mL of PBS until use. The lungs were homogenized and freeze-thawed three times. Serial dilutions were plated on confluent CV1 cells, and after 24 hours of culture at 37°C, the plates were stained with crystal violet, and plaques were counted.

### Disease score

Mice intranasally infected with VACV were weighed daily until animals lost more than 25% of the initial weight when they were sacrificed using CO_2_ overdose exposure, according to ethics guidelines. Symptoms observed daily to determine disease score were as follows: 0, no signs of disease, active, strong, curious, quick movements; 1, low grade: less active with occasional interruptions of activity, reduced alertness but appropriate response, slightly hunched; 2, medium grade: slow, drowsy, moves with difficulty, limited and delayed, hunched; 3, high grade: lethargic, immobile, severely hunched; 4, end point: death.

### Histopathological studies

Lungs, livers, and spleens were harvested without perfusion and were embedded in paraffin wax (253211, PanReac AppliChem). Serial 5 µm sections were stained with H&E (hematoxylin 75290, PanReac AppliChem; eosin 102439, Merck) or with PAS.

### Glucose, hemograms, and circulating tissue damage markers

Glucose levels were determined in blood drops from tails using Accu-Chek Aviva detector (6453970037, Roche). At sacrifice, blood samples were collected in EDTA tubes (1591126, EVEREST) by heart puncture, and 400 µL was sent to DYNAMIMED (Madrid) for analysis.

### Quantification of total circulating T3 and T4

Serum plasma was obtained by centrifugation of blood samples for 10 min at 6,000 rpm. T3 and T4 were measured using the Mouse Triiodothyronine (T3) ELISA kit (ab285259, Abcam) and Mouse Thyroxine (T4) ELISA kit (ab285258, Abcam), respectively. Assays were performed according to the manufacturer’s instructions, including a standard curve with a range of 0–7.5 µg/mL T3 or 0–25 µg/dL T4. The absorbance was measured in the BioTek EL340 Microplate Reader at 450 nm and subsequently converted to the corresponding concentration.

### Flow cytometry for immune cell identification and cytokine analysis

Spleen was dissociated with the GentleMACS dissector and mouse spleen dissociation kit (130-095-926, Miltenyi), according to the manufacturer instructions. For lung cell dissociation, the mouse lung dissociation kit was used (130-095-927, Miltenyi). Both cell suspensions were filtered through a 70 µm cell strainer and pelleted by centrifugation for 5 min at 300* × g*. Bone marrow cells were isolated from femurs cut at the ends by gentle centrifugation at 600 *× g* for 1 min and collected in 100 µL of FACS buffer (PBS, 2% FBS, and 5 mM EDTA). To lyse erythrocytes, pelleted cells were resuspended in 1 mL Versalyse lysing solution (A09777, Beckman Coulter). Two minutes later, 3 mL of FACS buffer was added, and cells were centrifuged and washed. The following markers were used to identify the different cell types from various tissues in spleen: B cells (B220^+^CD3^-^), T CD4^+^ cells (CD3^+^CD4^+^B220^-^), T CD8^+^ cells (CD3^+^CD8^+^B220^-^), NK cells (NKp46^+^B220^-^CD3^-^), neutrophils (CD11b^+^Ly6G^+^), RPMs (CD11b^+/low^Ly6G^-^F4/80^high^), and inflammatory monocytes (Ly6C^+^CD11B^+^Ly6G^-^); gating strategy is shown in Fig. S8; in lung: leukocytes CD45^+^ cells, neutrophils (CD45^+^Ly6G^+^), monocytes (CD45^+^Ly6G^-^CD11c^low/+^CD11b^+^MHCII^-^CD64^low/-^), interstitial macrophages (CD45^+^Ly6G^-^CD11c^low^CD11b^+^MHCII^-^CD64^+^SYGLEC^-^), and AMs (CD45^+^Ly6G^-^CD11c^+^CD11b^+^MHCII^-^CD64^+^SYGLEC^+^); gating strategy is shown in Fig. S9; and in bone marrow: B cells (B220^+^CD3^-^), T cells (CD3^+^B220^-^), neutrophils (Ly6G^+^CD3^-^B220^-^), natural killer (NKp56^+^Ly6G^-^CD3^-^B220^-^), and macrophages (F4/80^+^CD11b^+^Ly6G^-^CD3^-^B220^-^); gating strategy is shown in Fig. S10. The antibodies used to identify are listed in Table S1. Cytokines were determined using the LEGENDplex Mouse Anti-Virus Response Panel kit (740621, BioLegend) according to the manufacturer’s instructions. Cells and cytokine samples were subjected to flow cytometry analysis on a Cytoflex S (Beckman and Coulter). Data were analyzed using the CytoExpert (Beckman and Coulter) or LEGENDplex v.8.0 (BioLegend) software.

### Transfer of AMs

One day before infection, AMs were obtained from 8- to 10-week-old hypothyroid female mice by repeated bronchoalveolar lavages via the trachea with 37°C PBS-EDTA, as described previously (49, 50). The cells were centrifuged and analyzed by flow cytometry, which confirmed a viability and purity of approximately 95% of viable AMs. Subsequently, 0.5 × 10^6^ of these primary AMs in 20 µL PBS-EDTA were transferred to isoflurane-anesthetized hypothyroid recipient mice. For this, tracheas were surgically exposed and cannulated for instillation. Naive control mice received an equal volume of sterile PBS-EDTA.

### Statistical analysis

Two-tailed Student’s *t*-tests were used for comparisons between two groups. One-way ANOVA with *post-hoc* Tukey test was used to compare all pairs of columns from at least three different groups. Two-way ANOVA was used for curve comparison. The results are always expressed as means ± SEM. *P* values <0.05 were considered statistically significant. Significance is shown in the figures as *P < 0.05, **P < 0.01, and ***P < 0.001. Statistics were performed with GraphPad Prism 7.0 software.

## Data Availability

All data supporting the findings of this study are openly available in Digital.CSIC at https://doi.org/10.20350/digitalCSIC/17604.

## SUPPLEMENTAL MATERIAL

Supplemental material jvi.01294-25-s0001.pdf Figures S1 to S10; Table S1.

ASM does not own the copyrights to Supplemental Material that may be linked to, or accessed through, an article. The authors have granted ASM a non-exclusive, world-wide license to publish the Supplemental Material files. Please contact the corresponding author directly for reuse.
