# Supplementary material for: Thyroid hormone deficiency worsens outcomes in vaccinia virus infection
Source: J Virol. 2025 Nov 20;99(12):e01294-25. doi: 10.1128/jvi.01294-25 (PMC12724328; doi:10.1128/jvi.01294-25)
Supplement: Supplemental material — Figures S1 to S10; Table S1. [file jvi.01294-25-s0001.pdf]

Supplementary Material

Thyroid Hormones Deficiency Worsens Outcomes in Vaccinia Virus Infection.

Laura Notario[a,#], Erika Guerrero-Espinosa[b], Manuel Nistal[c], Pilar Lauzurica[a], Ana Aranda[b] and Susana Alemany[b,#]

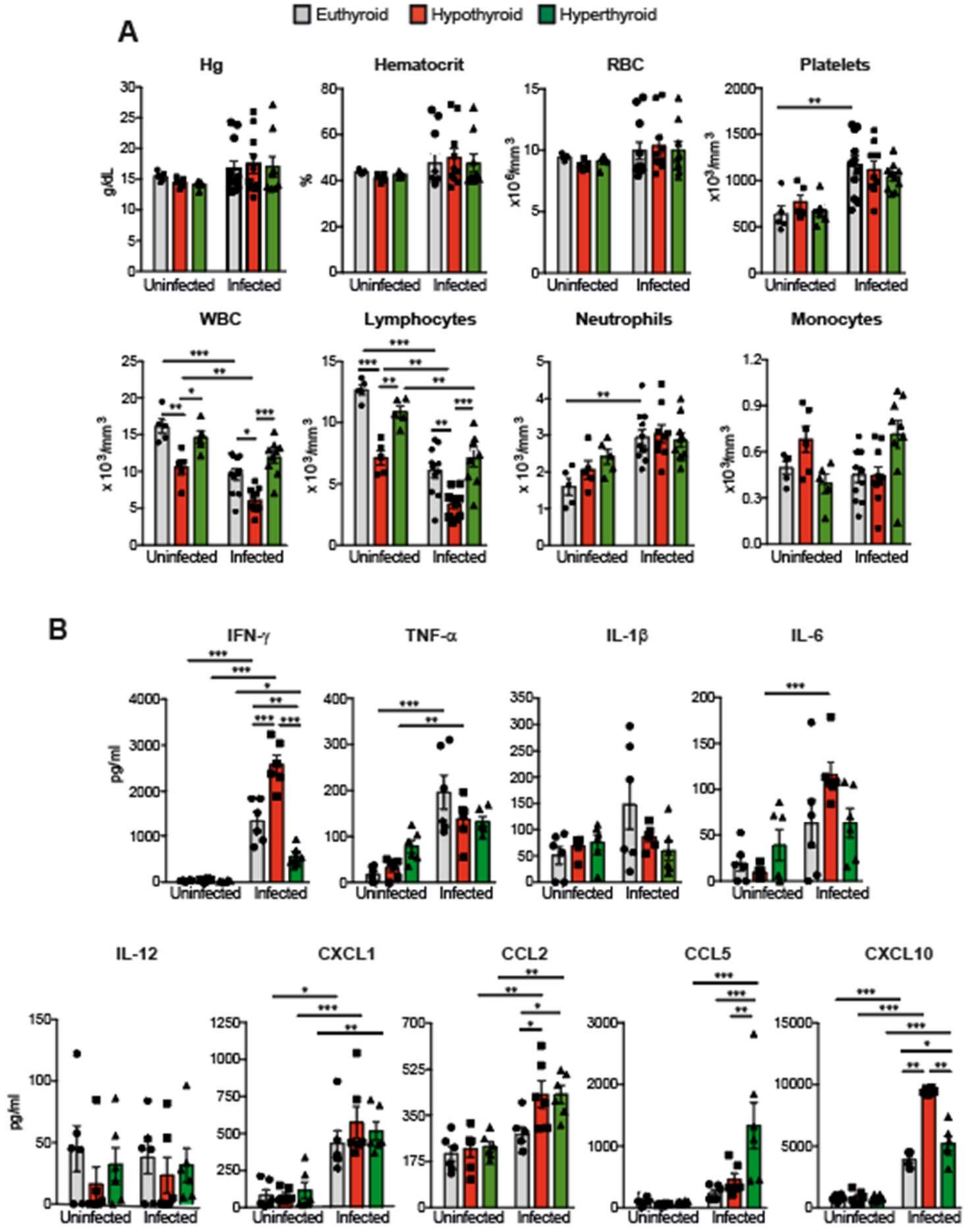

Supplemental Figure 1. Blood count and circulating cytokines after VACV infection. **(A)** Hemograms in euthyroid, hypothyroid and hyperthyroid mice at days 0 (uninfected) and 7 (infected) of intranasal inoculation with 5,700 PFU/ gram of body weight of VACV. Hemoglobin (Hg), Red blood cells (RBC), white blood cells (WBC). **(B)** Circulating levels of the indicated cytokines in the same groups of mice

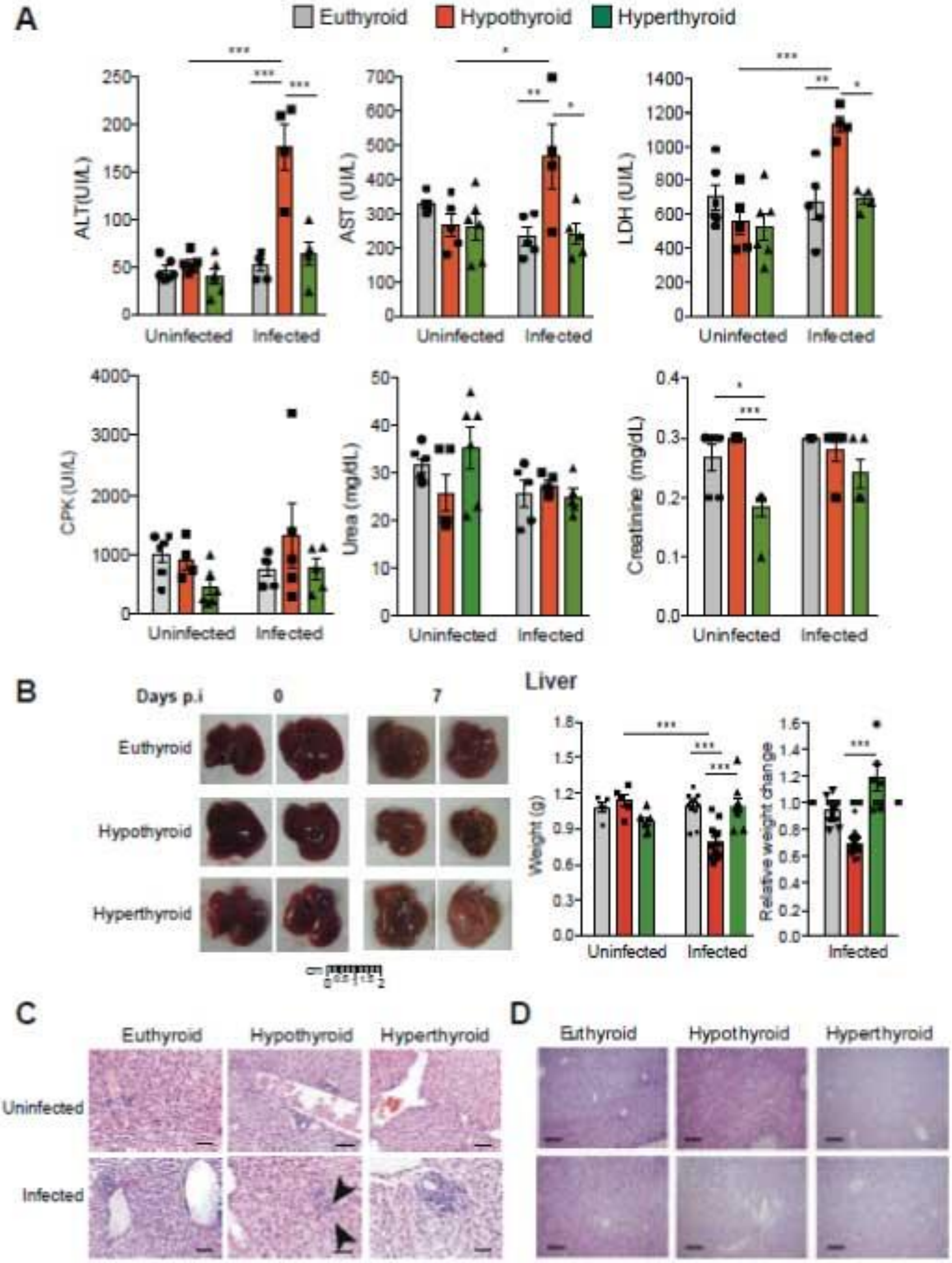

Supplemental Figure 2. Evaluation of tissue damage in euthyroid, hypothyroid and hyperthyroid mice in response to VAVC infection. **(A)** Circulating levels of organ damage markers in uninfected mice and at day 7 after intranasal infection with 5,700 PFU of VACV per gram of body weight. Alanine aminotransferase (ALT), aspartate aminotransferase (AST), lactate dehydrogenase (LDH), creatine phosphokinase (CPK). **(B)** Representative livers in euthyroid, hypothyroid and hyperthyroid treated as in A (Left panel). Liver weight of the different groups is shown in the middle panel. The relative change in liver weight of the infected groups with respect to the corresponding uninfected group is illustrated in the right panel. **(C)** Representative H&E staining of the uninfected and infected livers. Arrows show the presence of Councilman bodies in the infected hypothyroid animals. Scale bar 50 µm. **(D)** Representative liver Periodic acid–Schiff (PAS) staining of the same groups, suggesting glycogen loss in the hypothyroid mice after infection. Scale bar 100 µm.

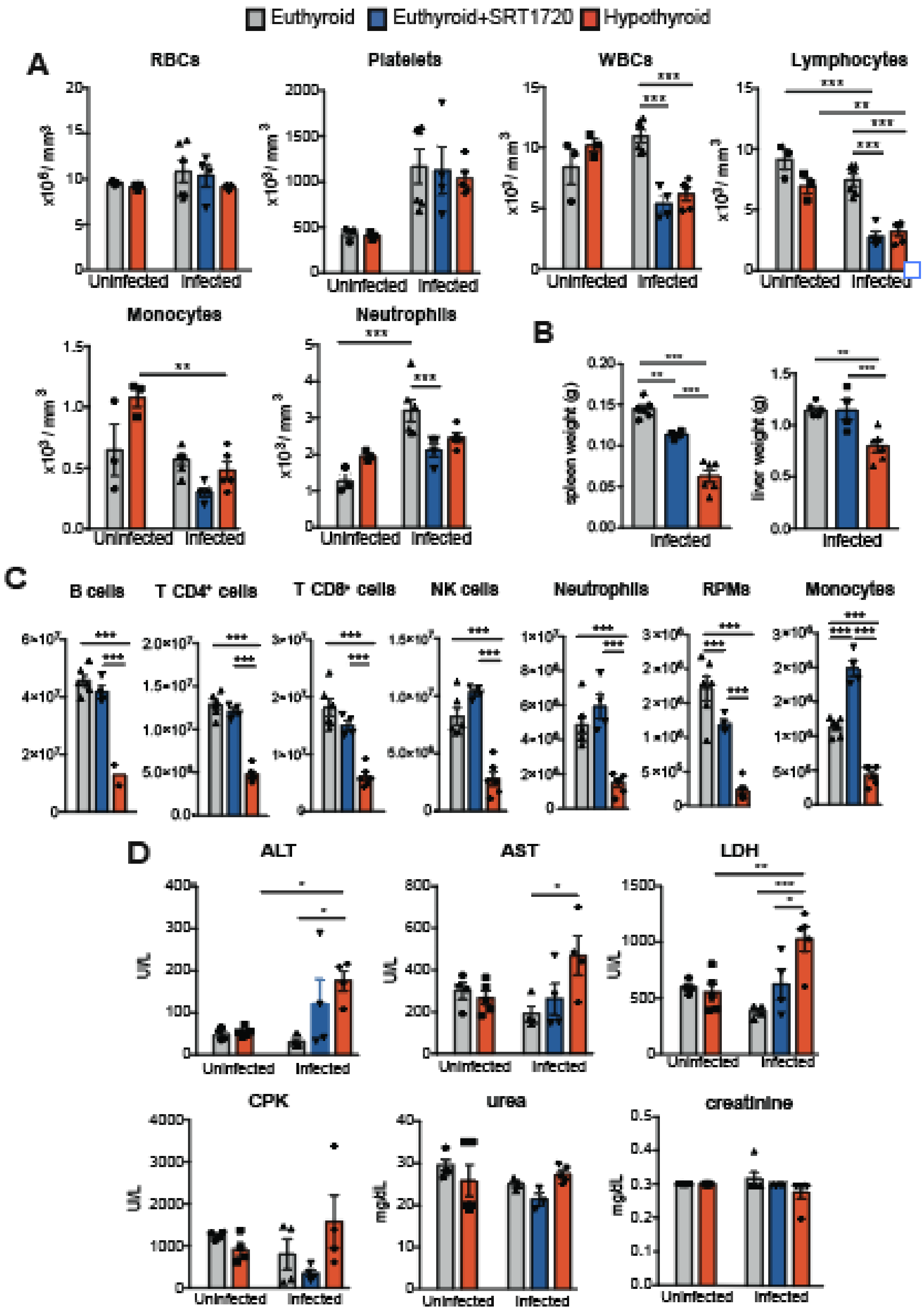

Supplemental Figure 3. Blood count, spleen changes and organ damage markers after VACV infection and treatment with SRT1720. (A) Hemograms in euthyroid mice treated daily with vehicle or SRT1720 (20 mg/kg) and in hypothyroid mice at days 0 to 7 p.i., with 5,700 PFU/ gram of body weight of VACV. Red blood cells (RBC), white blood cells (WBC). (B) Spleen and liver weights at day 7 p.i. of mice described in A. (C) Splenic cell populations determined by flow cytometry in the same groups of infected animals. B cells ($B220^{+}CD3^{-}$), T CD4+ cells ($CD3^{+}CD4^{+}B220^{-}$), T CD8+ cells ($CD3^{+}CD8^{+}B220^{-}$), natural killer cells (NK, $NKp46^{+}B220^{-}CD3^{-}$), neutrophils ($CD11b^{+}Ly6G^{+}$), red pulp macrophages (RPMs, $CD11b^{+/low}Ly6G^{-}F4/80^{high}$), and inflammatory monocytes ($Ly6C^{+}CD11B^{+}Ly6G^{-}$). Gating strategy is shown in Fig. S8. (D) Circulating levels of organ damage markers in the same groups of infected animals. alanine aminotransferase (ALT), aspartate aminotransferase (AST), lactate dehydrogenase (LDH); creatine phosphokinase (CPK).

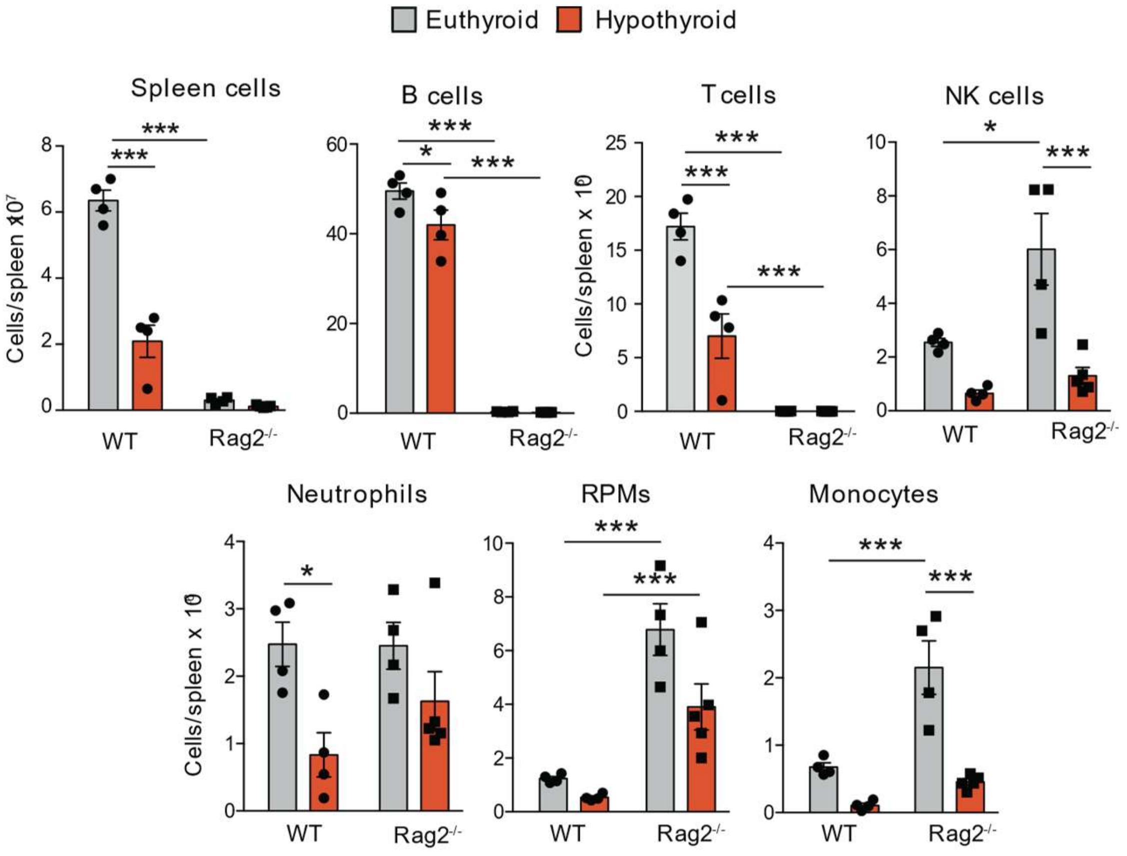

Supplemental Figure 4. **Splenic cells in euthyroid and hypothyroid Rag2$^{-/-}$ mice infected with VACV infection**. Total number of splenic cells, B cells (B220^{+}CD3^{-}), T CD4^{+} cells (CD3^{+}CD4^{+}B220^{-}), T CD8^{+} cells (CD3^{+}CD8^{+}B220^{-}), natural killer cells (NK, NKp46^{+}B220^{-}CD3^{-}), neutrophils (CD11b^{+}Ly6G^{+}), red pulp macrophages (RPMs, CD11b$^{+/low}$Ly6G^{-}F4/80^{high}), and inflammatory monocytes (Ly6C^{+}CD11B^{+}Ly6G^{-}) was determined by flow cytometry at day 0 and 7 after infection was determined by flow cytometry in euthyroid and hypothyroid wild type (WT) Balb/c and Rag2$^{-/-}$ Balb/c mice 4 days after intranasal infection with 5,700 PFU of VACV per gram of body weight. Gating strategy is shown in Fig. S8.

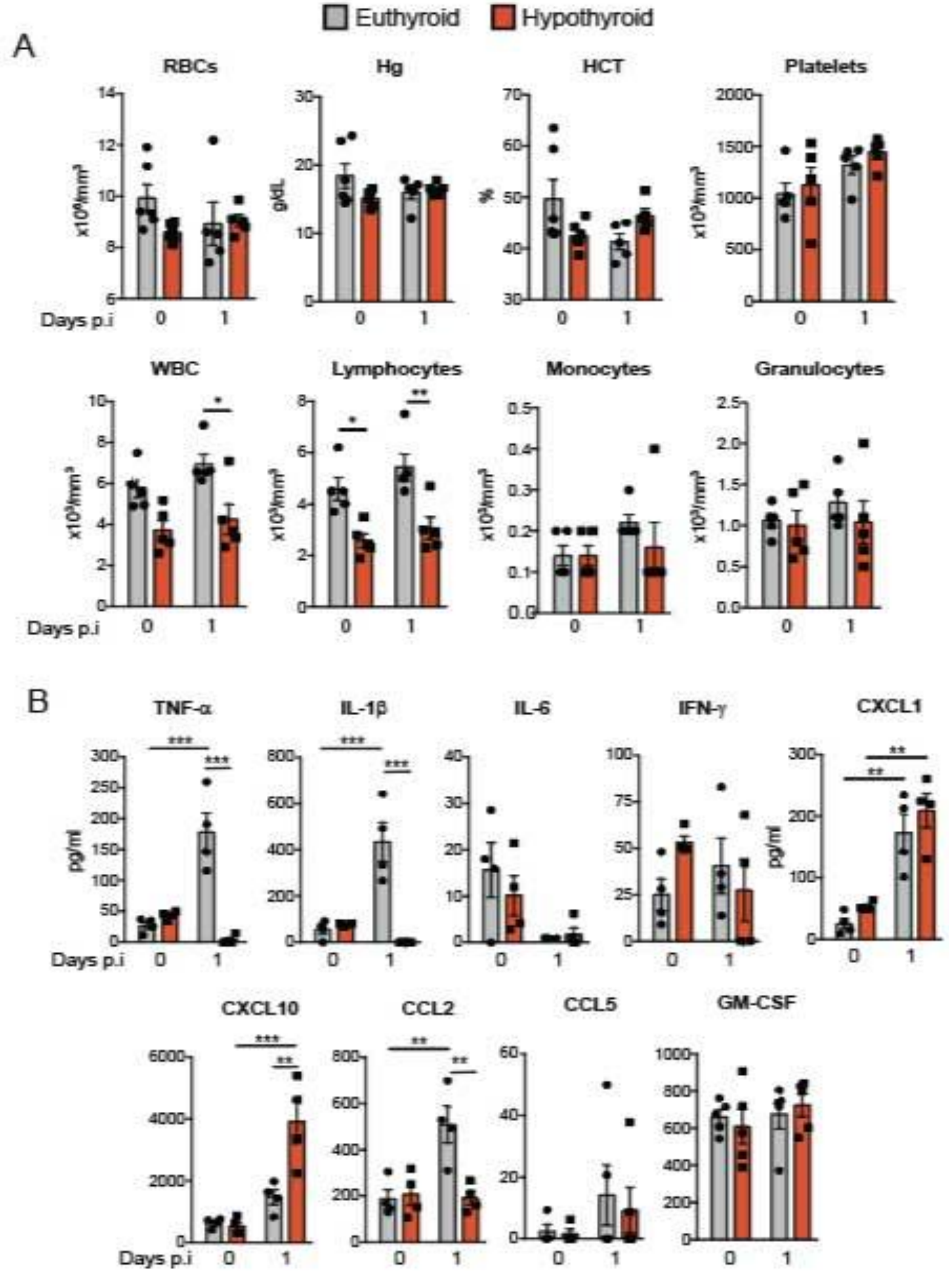

Supplemental Figure 5. Blood counts and circulating cytokines one day after VACV infection in euthyroid and hypothyroid mice. (A) Hemograms in euthyroid and hypothyroid mice at days 0 and 1 of intranasal inoculation of 5,700 PFU of VACV per gram of body weight. Red blood cells (RBCs), hemoglobin (Hg), hematocrit (HTC), white blood cells (WBC). **(B)** Levels of the indicated circulating cytokines from mice described in A.

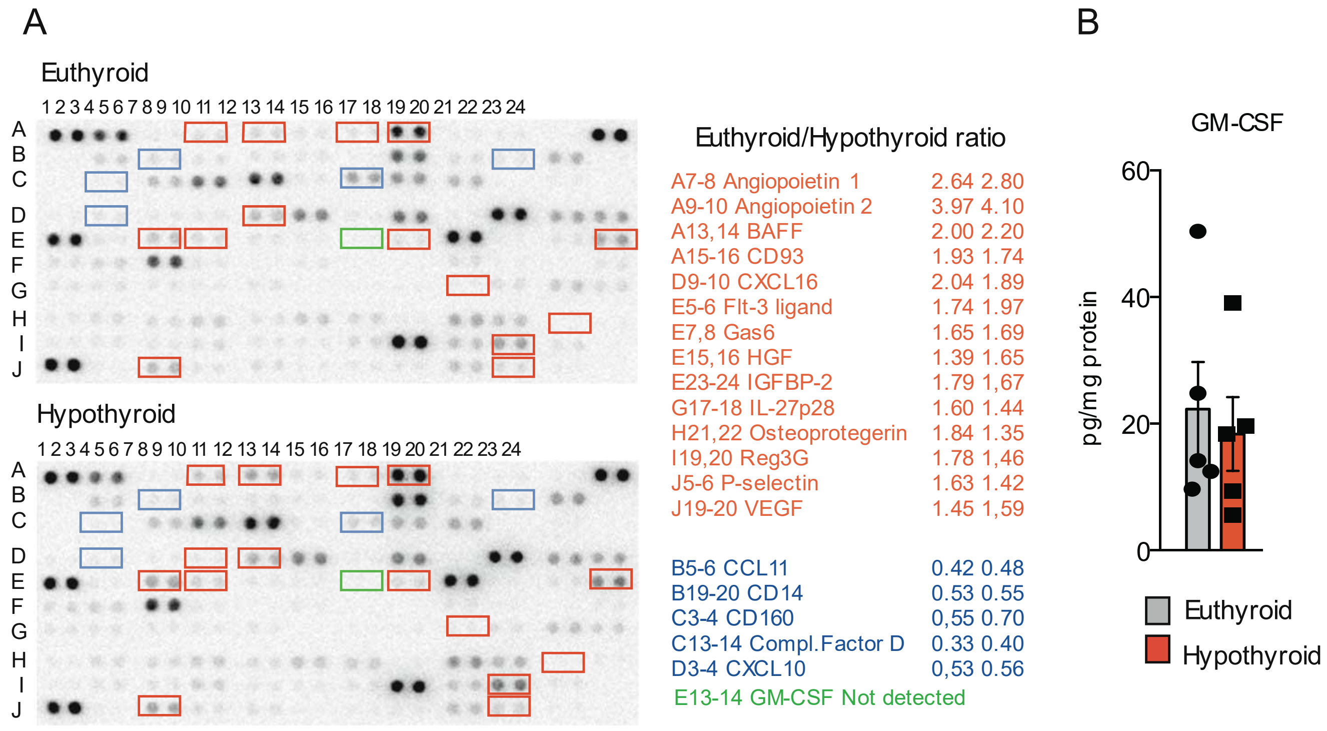

Supplemental Figure 6. Changes in the pattern of cytokine expression in the lungs of uninfected mice. (**A**) Blots of the Proteome Profiling Array Mouse XL Cytokine Array Kit (R&D Systems) in lungs of uninfected euthyroid and hypothyroid mice. Assays were performed in duplicate with lysates from 9 pooled lungs. Blots shown are from the 5 min. exposure and quantified with Image J. The list of lung proteins with euthyroid/hypothyroid values higher than 1.40 (red) and lower than 0.60 (blue) are shown at the right. (**B**) GM-CSF levels determined in individual lungs with LEGENDplex and flow cytometry.

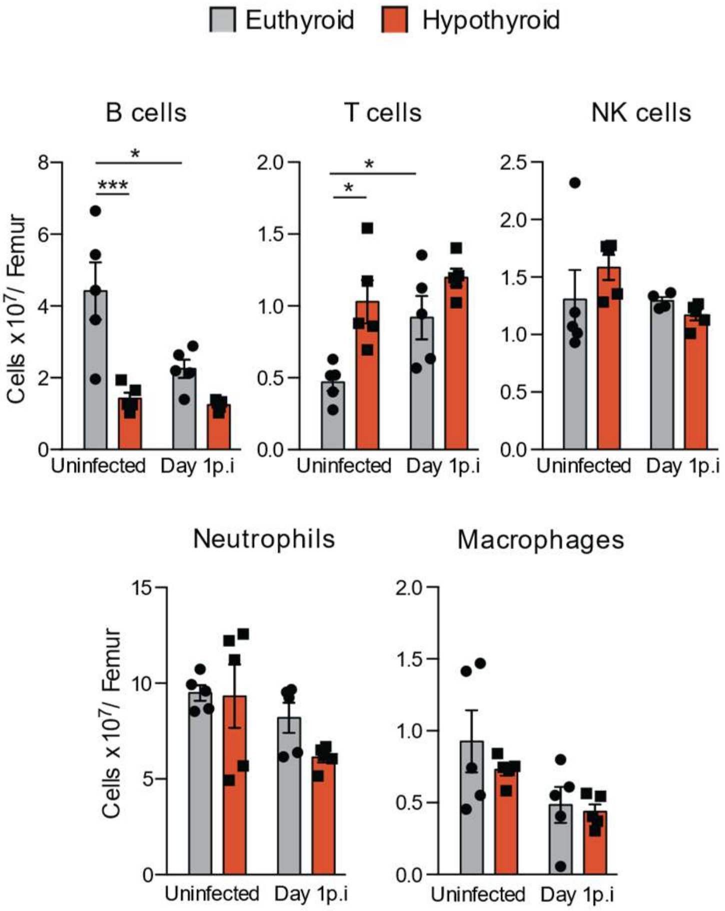

Supplemental Figure 7. **Bone marrow immune cells in euthyroid and hypothyroid mice after VACV infection.** The number of hematopoietic cells in the femurs of euthyroid and hypothyroid mice was quantified by flow cytometry at days 0 and 1 following intranasal with 5,700 PFU of VACV per gram of body weight B cells ($B220^{+}CD3^{-}$), T cells ($CD3^{+}B220^{-}$), neutrophils ($Ly6G^{+}CD3^{-}B220^{-}$), natural killer ($NKp56^{+}Ly6G^{-}CD3^{-}B220^{-}$), and ($F4/80^{+}CD11b^{+}Ly6G^{-}CD3^{-}B220^{-}$) by flow cytometry. The gating strategy is shown in Fig. S10.

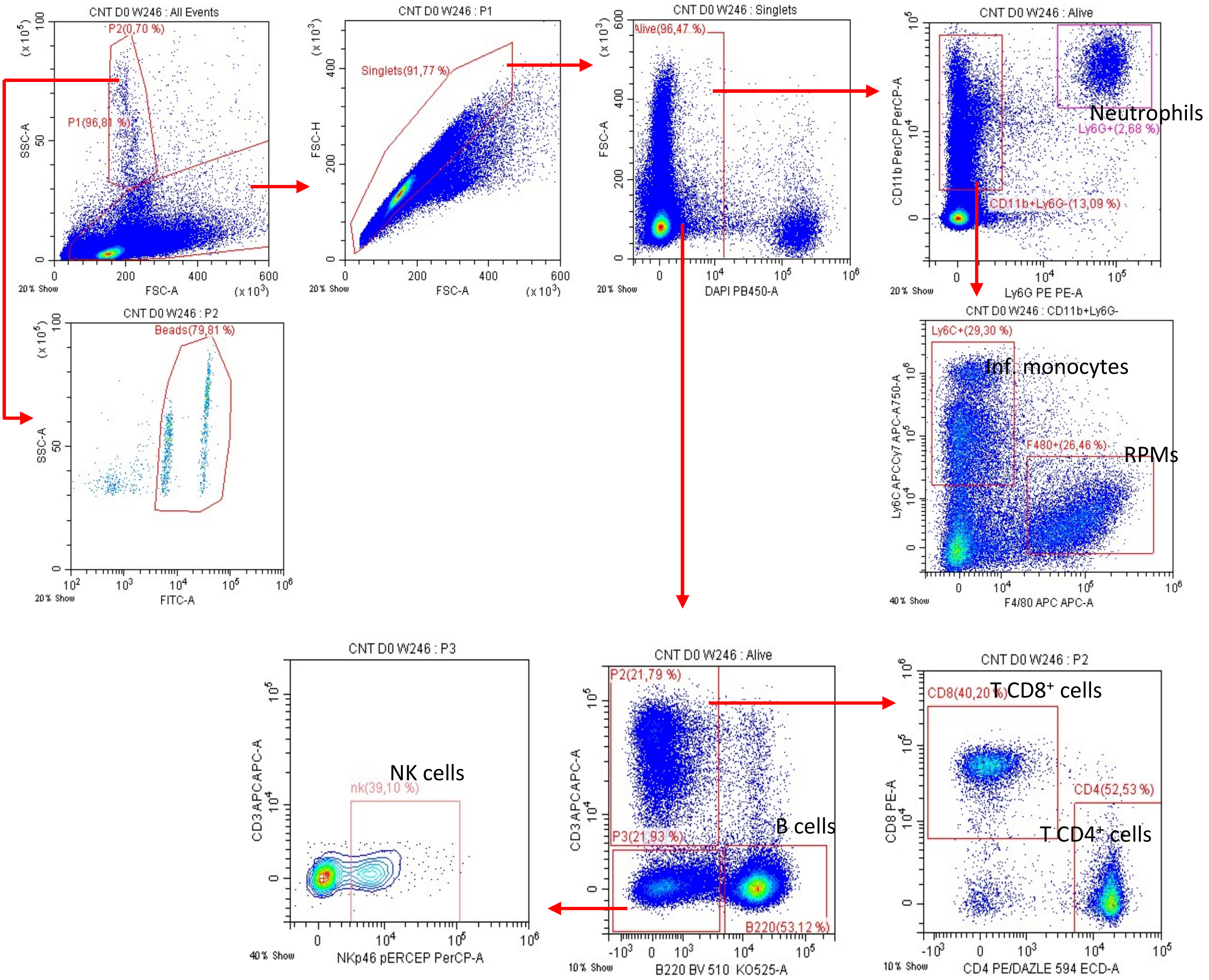

Supplemental Figure 8. **Gating strategy of splenic cell populations**. Identification strategy of splenic B cells ($B220^{+}CD3^{-}$), T $CD4^{+}$ cells ($CD3^{+}CD4^{+}B220^{-}$), T $CD8^{+}$ cells ($CD3^{+}CD8^{+}B220^{-}$), natural killer cells (NK, $NKp46^{+}B220^{-}CD3^{-}$), neutrophils ($CD11b^{+}Ly6G^{+}$), red pulp macrophages (RPMs, $CD11b^{+/low}Ly6G^{-}F4/80^{high}$), and inflammatory monocytes ($Ly6C^{+}CD11B^{+}Ly6G^{-}$).

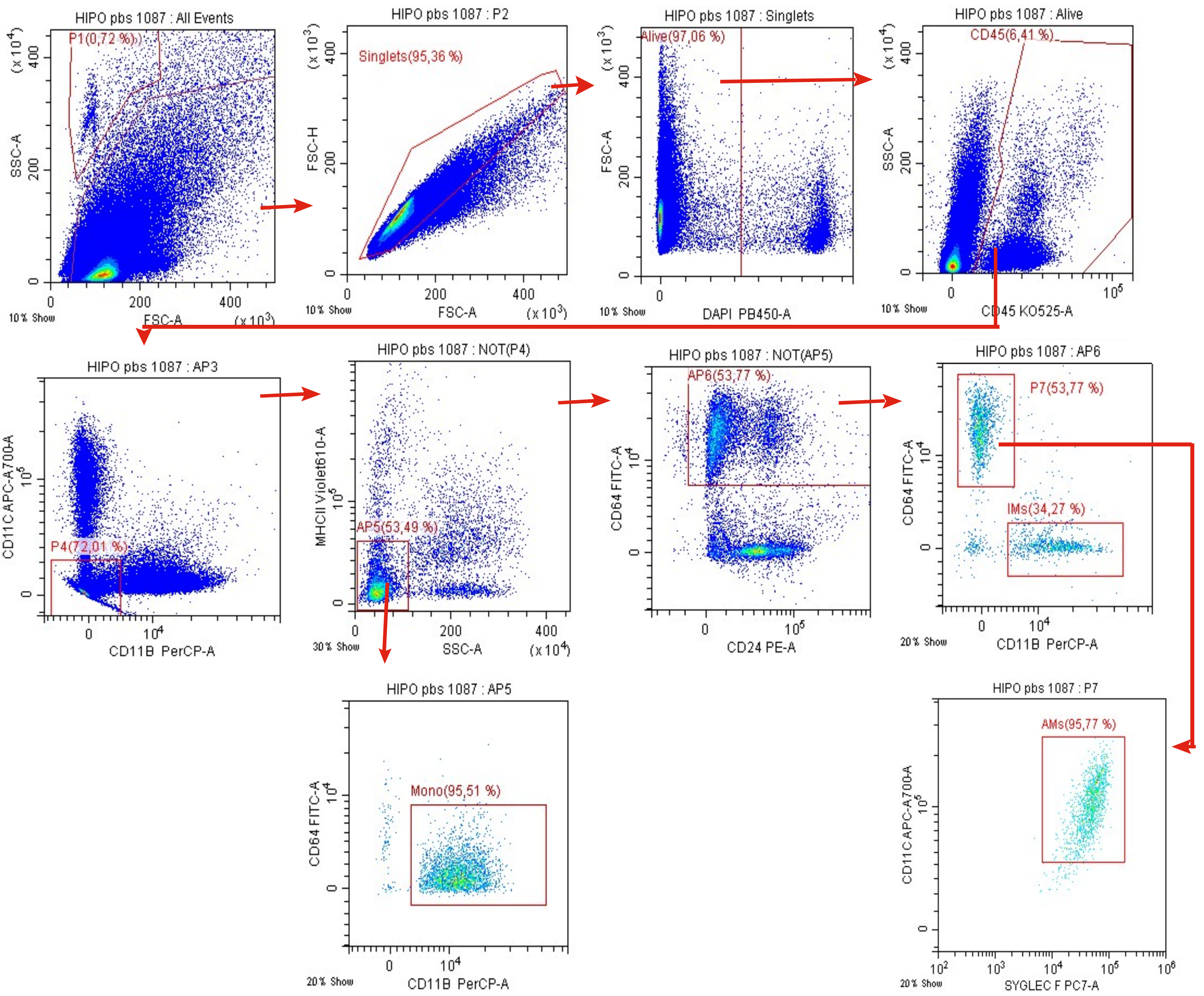

Supplemental Figure 9. **Gating strategy of lung cell populations.** Identification strategy of lung leukocytes $CD45^+$ cells, neutrophils ($CD45^+Ly6G^+$), monocytes ($CD45^+Ly6G^-CD11c^{low/+}CD11b^+MHCII^-CD64^{low/-}$), interstitial macrophages (IMs, $CD45^+Ly6G^-CD11c^{low}CD11b^+MHCII^-CD64^+SYGLEC^-$), and AMs ($CD45^+Ly6G^-CD11c^+CD11b^+MHCII^-CD64^+SYGLEC^+$) by flow cytometry.

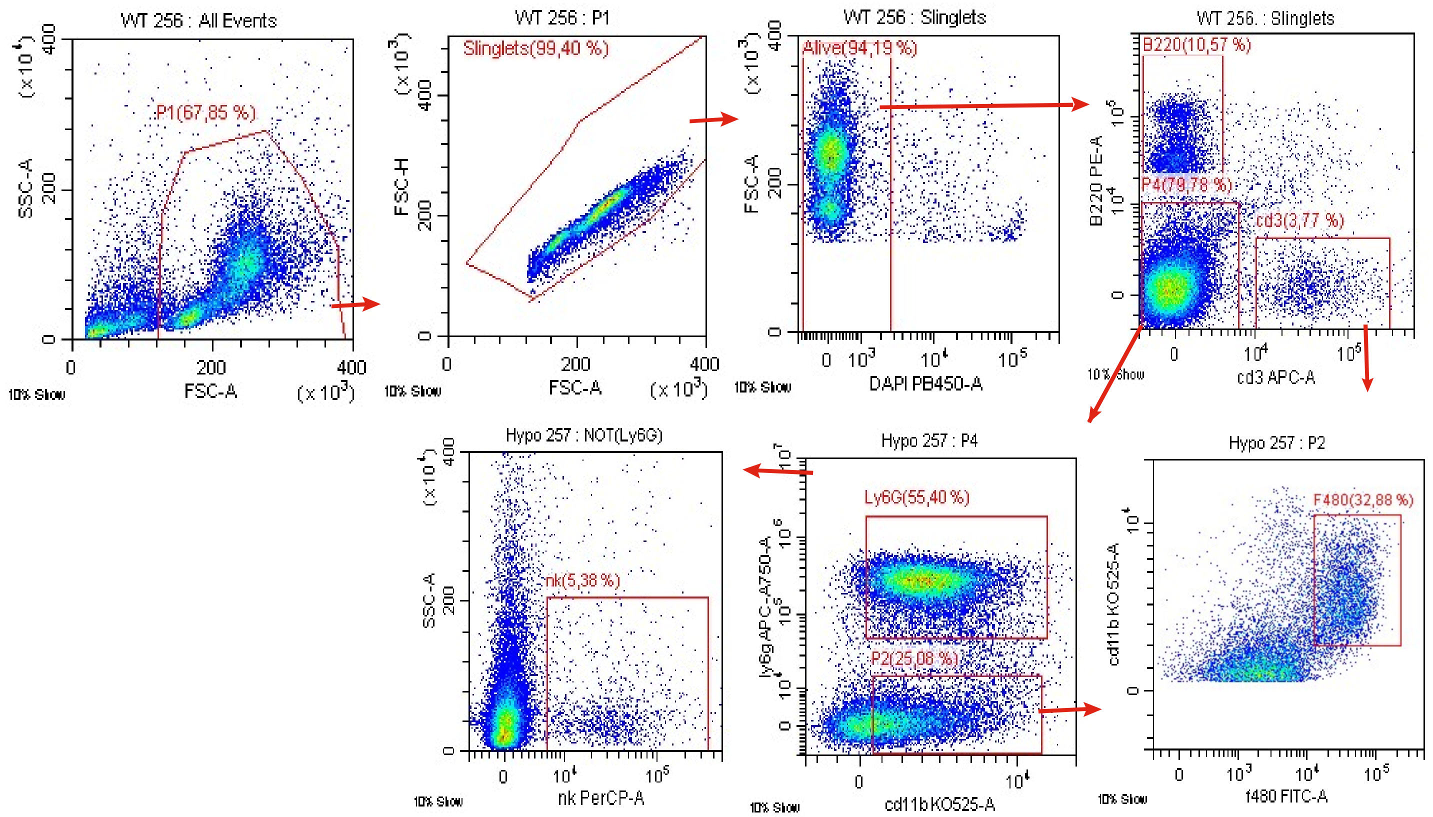

Supplemental Figure 10. **Gating strategy of bone marrow cell populations.** Identification strategy of bone marrow cells by flow cytometry. B cells ($B220^{+}CD3^{-}$), T cells ($CD3^{+}B220^{-}$), neutrophils ($Ly6G^{+}CD3^{-}B220^{-}$), natural killer ($NKp56^{+}Ly6G^{-}CD3^{-}B220^{-}$), and macrophages ($F4/80^{+}CD11b^{+}Ly6G^{-}CD3^{-}B220^{-}$).

Supplementary Table 1

Antigen	Fluorophore	Clone	Source
B220	PE	RA3-6B2	Biolegend
B220	BV 510	RA3-6B2	Biolegend
CD11b	BV510	M1/70	Biolegend
CD11b	PerCP	MI-70	BD Bioscience
CD11c	AF700	N418	Biolegend
CD24	PE	M1/69	Biolegend
CD3	APC	145-2C11	Biolegend
CD4	PE/Dazzle 594	GK1.6	Biolegend
CD45	BV510	30-F11	Biolegend
CD64	FITC	X54-5/7-1	Biolegend
CD8	PE	53-6.7	Biolegend
DAPI		62248	Thermofisher
F4/80	Fitc	BM8.1	Biolegend
F4/80	APC	BM8.1	Biolegend
Ly6C	APC-Cy7	HK1.4	Biolegend
Ly6G	APC-A750	RB6-8C5	Biolegend
Ly6G	PE	130-107-913	Miltenyi
MHC II	BV605	M5/114.15.2	Biolegend
Nkp46	PerCP	29A1.4	BD Bioscience
SIGLEC-F	Pecy7	S17007L	Biolegend
